# The Added Value and the Efficacy of Preoperative Physiotherapy on Degenerative Diseases of the Lumbar Spine: A Systematic Review

**DOI:** 10.1002/pri.70214

**Published:** 2026-04-17

**Authors:** Manna Jacopo, Fanigliulo Ciro, Giardulli Benedetto, Testa Marco, Andreoletti Federico

**Affiliations:** ^1^ Department of Neurosciences, Rehabilitation, Ophthalmology, Genetic and Maternal Infantile Sciences (DINOGMI) University of Genova ‐ Campus of Savona Savona Italy; ^2^ School of Health and Society Centre for Human Movement and Rehabilitation University of Salford Salford UK

**Keywords:** degenerative diseases of the lumbar spine, lumbar surgery, physiotherapy, spine surgery, systematic review

## Abstract

**Background and Purpose:**

Degenerative Diseases of the Lumbar Spine (DDLS) are typically managed with conservative treatments, while surgery is reserved for cases with severe motor and/or sensory deficits. Preoperative physiotherapy has been shown to improve postoperative outcomes in shoulder, hip, and knee surgeries. However, its effects on the lumbar spine have been poorly investigated. This systematic review aimed to evaluate the efficacy of preoperative physiotherapy on pain, disability, and hospitalisation in individuals with DDLS.

**Methods:**

A literature search was conducted in MEDLINE, Cochrane CENTRAL, Scopus, PEDro and EMBASE. Two reviewers independently screened the studies, extracted the data, and assessed the risk of bias. We included randomised controlled trials (RCTs) in which preoperative interventions were delivered by physiotherapists and compared to usual care or other interventions. We conducted meta‐analyses on primary outcomes.

**Results:**

We screened 9.803 studies and included 6 with 5 different study populations. All studies were RCTs that evaluated the efficacy of preoperative physiotherapy compared with usual care in pain and disability. The preoperative interventions were heterogeneous, varying between therapeutic exercise, cognitive‐behavioural therapies, and multimodal interventions. The meta‐analyses showed no difference between interventions. All studies had a high risk of bias.

**Conclusion:**

The effects of preoperative physiotherapy, compared with usual care, on pain, disability, and hospitalisation among patients with DDLS, are minimal. However, the risk of bias of the included studies and the certainty of evidence, based on the GRADE approach, were very low. Future high‐quality, low‐bias trials are needed to clarify its effectiveness and identify patient subgroups that may benefit the most.

**Abbreviations**
ACT: Acceptance and Commitment Therapy
CBT: Cognitive Behavioural Therapy
CFT: Cognitive Functional Therapy
CT: Computerized Tomography
DDLS: Degenerative Diseases of the Lumbar Spine
FABQ: Fear Avoidance Beliefs Questionnaire
HADS: Hospital Anxiety and Depression Scale
LBP: Low Back Pain
LBPRS: Low Back Pain Rating Scale
MRI: Magnetic Resonance Imaging
NPRS: Numeric Pain Rating Scale
ODI: Oswestry Disability Index
PNE: Pain Neuroscience Education
RCT: Randomised Controlled Trial
RMQ: Roland Morris Questionnaire
SES: Self‐Efficacy Scale
SF‐36: Short Form‐36
VAS: Visual Analogue Scale

## 1 Background

Degenerative Diseases of the Lumbar Spine (DDLS) encompass a wide range of degenerative conditions of the lumbar district and surrounding structures (Gallucci et al. 2007). The most common DDLS are stenosis, spondylolysis, spondylolisthesis and disk displacements. Specific pathologies and trauma, but especially overuse and repeated microtrauma, can lead to DDLS (Gallucci et al. 2007). In most cases, the management of these conditions is conservative, including manual therapy, therapeutic exercise, and pharmacological treatment (Apeldoorn et al. 2024; Chan et al. 2019; Chiu et al. 2015; Merskey 1986).

When conservative management for DDLS is not sufficient to improve symptoms, surgery can be an effective strategy, especially in the presence of severe symptoms and progressive neurological involvement (Zhong et al. 2017; Diwan et al. 2019; Kawakami et al. 2023). Absolute indications for surgery include cauda equina syndrome and foot drop (Yoon and Koch 2021). The most common surgical procedures are discectomy, decompression, and/or fusion (Machado et al. 2016; Mobbs et al. 2015; Rasouli et al. 2014).

Current evidence supports the effectiveness of physiotherapy following lumbar spine surgery, particularly in cases of disc herniation and lumbar stenosis (McGregor et al. 2013; Pourahmadi et al. 2022). High‐intensity therapeutic exercise has been shown to significantly reduce pain and disability in the postoperative stage (Pourahmadi et al. 2022). The recommended interventions include cognitive behavioural therapy (CBT), cardiovascular training, neurodynamic techniques, and motor control exercises. In contrast, passive interventions such as manual therapy, electrotherapy, and bracing show limited effectiveness (Bogaert et al. 2022; Madera et al. 2017; Temporiti et al. 2022). Pharmacological management may include NSAIDs or epidural corticosteroid injections for short‐term symptom relief, while gabapentinoids, benzodiazepines, and oral corticosteroids are generally discouraged due to limited efficacy (Kawakami et al. 2023; *Overview | Low Back Pain and Sciatica in over 16s* 2016).

However, the effectiveness of physiotherapy in the preoperative phase of DDLS remains uncertain, even though preoperative physiotherapy has benefits in other surgical populations, leading to benefits such as improved function, quality of life, and muscle strength compared to standard care (de Almeida et al. 2021; Punnoose et al. 2023). As an example, in procedures such as total joint replacements or rotator cuff repair, preoperative exercise has been linked to better postoperative strength, reduced pain, greater mobility, and sustained improvements up to 1 year after surgery (de Almeida et al. 2021; Shaarani et al. 2013). A recent systematic review tried to investigate the effects of preoperative physiotherapy in patients with DDLS and found no significant advantage over usual care based on the limited number of available studies (Janssen et al. 2021). Notably, this review includes studies employing designs other than randomised controlled trials (RCTs), and in some cases, the interventions were not delivered by physiotherapists. Another systematic review aimed to determine whether a preoperative education session improves clinical, psychological and economic outcomes in elective spinal surgery (Burgess et al. 2019). The authors found no differences in quality of life, return to work, physical indicators, or postoperative complications.

Hence, the following systematic review aimed to evaluate the efficacy of preoperative physiotherapy on pain, disability, and hospitalisation in individuals with DDLS.

## 2 Materials and Methods

The protocol of this systematic review was registered in Prospero (CRD42024542242) (Booth et al. 2012). This systematic review adhered to Preferred Reporting Items for Systematic Reviews and Meta‐Analyses (PRISMA) (Tricco et al. 2018) and followed the recommendations of the Cochrane Handbook for Systematic Reviews (Higgins et al. 2011).

### 2.1 Search Strategy

A systematic search was conducted in the following databases: MEDLINE (via PubMed), CENTRAL (via the Cochrane Library), PEDro, EMBASE, and Scopus, as suggested by the Cochrane Handbook for Systematic Reviews (Higgins et al. 2011). The search strategy was based on the Population Intervention Comparison Outcome (PICO) acronym and has been created by combining with the boolean operators ‘AND’ and ‘OR’ the keywords ‘physical therapy’, ‘prehabilitation’, ‘pre‐surgery physiotherapy’, ‘lumbar region’, ‘spinal surgery’, ‘spinal stenosis’, ‘intervertebral disk displacement’ and their synonyms and abbreviations. It has been adapted to the different databases and has been conducted until September 2025. Search strategies are reported in Supporting Information S1: Appendix S1. A search of the grey literature was also conducted using the SIGLE (System for Information on Grey Literature) database. To find other possible relevant studies, the bibliography of the included studies has been analysed. There were no language or time restrictions.

### 2.2 Eligibility Criteria

#### 2.2.1 Types of Study

Only RCTs evaluating the effects of preoperative physiotherapy on pain, disability, and hospitalisation in patients with DDLS were included. Studies were limited to RCTs conducted on human subjects with no date or language restrictions.

#### 2.2.2 Population

Adult individuals (> 18 years old) with DDLS (e.g., intervertebral disk displacement, spinal stenosis, spondylolysis/spondylolisthesis) diagnosed by history and physical examination and confirmed by imaging (MRI or TC), and currently waiting for upcoming spinal surgery. Exclusions were made for specific conditions such as cancer, spinal fractures, infections, and mental disorders.

#### 2.2.3 Intervention

Studies were considered eligible if at least one group of participants received either preoperative physiotherapy alone or combined with other therapies (e.g., pharmacological treatment, psychological treatment). Preoperative physiotherapy consisted of various interventions delivered by physiotherapists, including manual therapy, exercise, electrotherapies and education interventions (CBT; Pain Neuroscience Education, PNE; Cognitive Functional Therapy, CFT; Acceptance and Commitment Therapy, ACT), since physical therapists are trained to provide some psychological intervention too (Farrell et al. 2023). In the case of transversal techniques, such as PNE, it was considered a physiotherapy intervention in case it was provided by a physiotherapist. The only information about surgical procedures was not considered as an intervention, neither by booklets nor by healthcare professionals.

#### 2.2.4 Comparison

Studies were included if control groups received no intervention (physical therapy vs. no intervention), only information (via booklets or from healthcare professionals) regarding surgical procedures, or if physiotherapy was provided as an adjunct to another treatment (physical therapy + Treatment A vs. Treatment A).

#### 2.2.5 Outcome

The primary outcomes included pain (Numeric Pain Rating Scale, NPRS; Visual Analogue Scale, VAS; McGill Pain Questionnaire), hospitalisation, and adverse events. Secondary outcomes included disability (Oswestry Disability Index, ODI; Roland Morris Questionnaire, RMQ), quality of life (Short Form 36, SF‐36), fear avoidance (Fear Avoidance Belief Questionnaire, FABQ), self‐efficacy (Self‐Efficacy Scale, SES), and anxiety and depression (Hospital Anxiety and Depression Scale, HADS). No follow‐up limits were set.

### 2.3 Selection Process

Studies were uploaded onto the Rayyan QRCI website (Ouzzani et al. 2016). After duplicate removal, two reviewers independently screened titles and abstracts for eligibility. Full texts were read when necessary. Discrepancies were resolved through discussion between two reviewers (F.C. and M.J.), and a third reviewer (A.F.) was consulted if needed. Full‐texts for the last screening were retrieved from the Library Service of the University of Genoa. Studies identified as substudies of the included trials were used to complete outcome measures.

### 2.4 Data Extraction

Two review authors (F.C. and M.J.) independently extracted the following data from all eligible papers using a standard data extraction form: study characteristics (author, title, design, country, year of publication), population characteristics (number, age, gender and/or sex assigned at birth), intervention and control characteristics, outcome measures and follow‐up. Disagreements were resolved by a consensus or with a third author (A.F.). Study authors were contacted for missing or incomplete data.

### 2.5 Risk of Bias

The quality of the included studies was assessed independently by two review authors (F.C. and M.J.) using the Cochrane Risk‐of‐Bias tool for RCTs (RoB 2.0 tool) (Higgins et al. 2011). This tool evaluates five domains: randomisation process, deviations from intended intervention, missing data outcome, measurement of the outcome, and selection of the reported result. Each domain has been evaluated as ‘low risk’, ‘high risk’, or ‘some concerns’. Any disagreement during the quality assessment process was identified and resolved through discussion with a third author (G.B.).

### 2.6 Data Analysis and Synthesis

Review Manager 5.3 (RevMan‐Copenhagen: The Nordic Cochrane Center, The Cochrane Collaboration, 2014) software was used for calculations. This analysis was performed using a comparison of means and standard deviations, or the mean differences (SMDs) with 95% confidence intervals of intervention groups with control groups, or medians and interquartile ranges (IQRs) when possible. Meta‐analyses were performed using the random‐effect model. Forest plots were used to display the results of the meta‐analysis graphically. Heterogeneity among studies was explored by visual inspection of the 95% CI of the forest plots, by *X* ^2^ test (if *X* ^2^ > degrees of freedom; significance level < 0.1), and *I* ^2^ statistic (considered acceptable below 50%, above 50% caution should be used in the interpretation of the meta‐analysis). The analysis compared values measured at baseline, after treatment and, where applicable, during subsequent follow‐ups over time. Values were discussed considering the minimal clinically important difference (MCID) of each outcome to identify their significance and importance in terms of concreteness and clinical relevance. The Grading of Recommendations Assessment, Development and Evaluation (GRADE) was used to appraise and report certainty of the evidence of the systematic review for each included outcome (Guyatt et al. 2008). The downgrading process was based on five domains: study limitations (e.g., risk of bias), inconsistency (e.g., heterogeneity between study results), indirectness of evidence (generalisability and transferability, e.g., short‐term follow‐up), imprecision (e.g., small sample size), and reporting bias (e.g., publication bias).

## 3 Results

### 3.1 Study Selection

**FIGURE 1:**
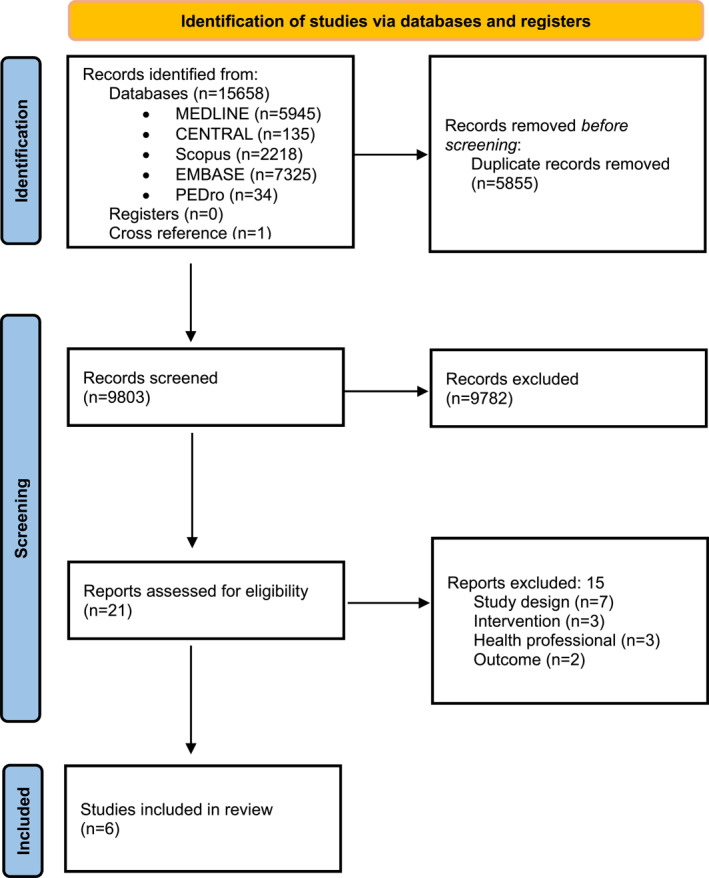
PRISMA 2020 flow chart.

The search strategy identified 15.658 records. After removing duplicates (*n* = 5.855), we assessed the remaining records by title and abstract. During the full‐text screening, the studies were reduced to 21. Full texts of 21 studies were screened for inclusion, and 6 studies (Lindbäck et al. 2018; Lotzke et al. 2019; Louw et al. 2014, 2016; Nielsen et al. 2010; Rolving et al. 2015) met the eligibility criteria and were included in this systematic review. One of these (Louw et al. 2016) is a follow‐up of another included study (Louw et al. 2014). Two authors were contacted, and only one answered. The entire selection process is illustrated in Figure 1.

### 3.2 Characteristics of the Included Studies

The included studies were published between 2010 and 2019. Sample sizes ranged from 73 to 197 participants, and all trials included two study arms. Regarding geographical distribution, two studies were conducted in Sweden (Lindbäck et al. 2018; Lotzke et al. 2019), two in Denmark (Nielsen et al. 2010; Rolving et al. 2015), and one in the United States (Louw et al. 2014). The types of surgical interventions varied across studies: Lindbäck et al. (Lindbäck et al. 2018) did not specify the surgical procedure; Nielsen et al. (2010) included participants who underwent spinal fusion with or without decompression; Louw et al. (2014) focused on decompression surgery, and both Lotzke et al. (2019) and Rolving et al. (2015) involved participants who underwent spinal fusion.

All the studies included were RCTs. The total sample size was 545 individuals, 247 (45%) of whom were men and 298 (55%) women. The mean age could not be calculated because some studies lacked the necessary data: one study did not report the standard deviations for either the intervention or control group (Louw et al. 2014); another reported only age ranges instead of standard deviations (Nielsen et al. 2010); and one study reported individual‐level data without providing mean values for each group (Rolving et al. 2015). The characteristics of the studies are presented in Table 1.

**TABLE 1:** Study characteristics of the included studies.

Author and year	Study design	Country	Sample size (m, w)	Mean age (SD)	Intervention	Control
Lindbäck et al. (2018)	RCT	Sweden	197 (92; 105)	59,50 (12,5)	Patients received preoperative intervention twice a week for 9 weeks. The program included: (1) physiotherapy according to a treatment‐based classification (TBC; specific exercises and mobilisation, motor control exercises, or traction); (2) a tailor‐made general supervised exercise program; (3) a behavioural approach to reduce fear avoidance and increase activity level	Patients received standardised information from an orthopaedic surgeon about surgery, post‐surgery rehabilitation, and advice to stay active
Lotzke et al. (2019)	RCT	Sweden	118 (55; 63)	45,70 (8,3)	The preoperative physiotherapy phase started 8–12 weeks before surgery. Participants in the intervention group met the same physical therapist at a spine clinic for four 1‐h sessions before surgery, and 1 half‐hour session by telephone before surgery, and 1 half‐hour session by telephone 2 weeks after surgery. Each session had a predefined structure, a specific aim, and included several cognitive‐behavioural techniques	Conventional care is comprised of a single session with a physical therapist. In this, the patient should receive information about the postoperative mobilisation routine and be introduced to a core exercise program to be initiated the day after surgery. Furthermore, the patient should be encouraged to stay active and to start performing the recommended exercises before surgery
Louw et al. (2014, 2016)	RCT	USA	67 (31; 36)	md	The neuroscience Education (NE) session averaged 30 min. Patients were additionally provided with a preoperative NE booklet summarising the educational content of the preoperative NE session, including pictures, examples, and metaphors	Patients in the control group (UCG) received what constitutes ‘usual care’ regarding preoperative education from their respective surgeons and staff
Nielsen et al. (2010)	RCT	Denmark	73 (30; 43)	md	All intervention patients received a 6‐ to 8‐week, individualised, preoperative training program for exercise at home. The program focused on the improvement of muscle strength for the back and abdomen and included cardiovascular conditioning. The patients were informed and advised regarding cessation of smoking and harmful drinking before surgery. Fourteen days before the operation, the intervention group, eventually with relatives, met with the physiotherapist, and once again received information about the operation, postoperative mobilisation and rehabilitation. The evening before surgery, the intervention group supplemented their usual food intake with 200 mL protein‐rich drinks	The control group followed departmental routines. Once the surgeon took a decision on surgery, the surgeon informed the patient about the operation and advised them regarding cessation of smoking and harmful drinking before surgery. At admission, the anaesthesiologist informed the patient about anaesthesia and postoperative pain treatment, and the nurse in the surgical department informed the patient about the plan for postoperative rehabilitation in the hospital, including pain treatment, diet and physiotherapy following the operation day, aiming for discharge on the eighth postoperative day
Rolving et al. (2015)	RCT	Denmark	90 (39; 51)	md	In addition to the standard course of treatment, patients participated in six 3‐h group sessions (4 sessions preoperatively and 2 sessions postoperatively). The main topics covered were the interaction of cognition and pain perception, coping strategies, pacing principles, ergonomic directions, return to work, and details about the surgical procedure	Patients in the control group received the standard course of treatment, which involves preoperative information about the upcoming operation and the anaesthetic procedure, medication, and information about the postoperative rehabilitation and physical restrictions after surgery. Information was given by the operating surgeon, nurses, physiotherapists, and occupational therapists

Abbreviations: m = men; md = missing data; RCT = randomized controlled trial; SD = standard deviations; w = women.

### 3.3 Risk of Bias

**FIGURE 2:**
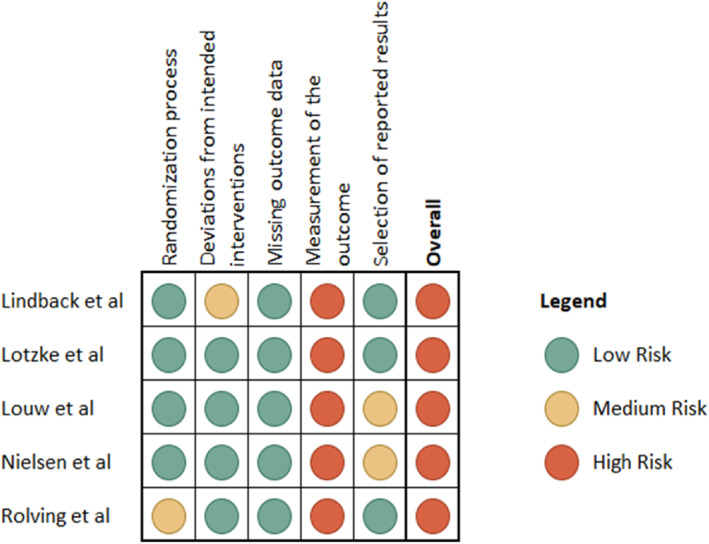
Risk of bias assessment.

The overall risk of bias was high for all the included papers due to the high risk identified in the fourth domain. Some studies presented some concerns in the randomisation process (Rolving et al. 2015), deviations from attended interventions (Lindbäck et al. 2018), and the selection of reported results (Louw et al. 2014; Nielsen et al. 2010). The risk of bias results are presented in Figure 2.

### 3.4 Primary Outcomes

We identified pain, hospitalisation, and adverse events as primary outcomes. All included studies assessed pain using measures such as the VAS (Lindbäck et al. 2018; Lotzke et al. 2019; Nielsen et al. 2010), the NPRS (Louw et al. 2014), and the Low Back Pain Rating Scale (LBPRS) (Rolving et al. 2015). These measures were reported for both low back pain and radiating leg pain. Hospitalisation and adverse events were not reported across the studies except for one study (Nielsen et al. 2010), which included this information in a table.

Only one study reported significant differences in back pain at pre‐surgery follow‐up (Lindbäck et al. 2018). Nielsen observed a significant reduction in hospitalisation in the intervention group. Three studies declared the absence of adverse events (Lindbäck et al. 2018; Lotzke et al. 2019; Rolving et al. 2015), and Nielsen did not find any difference between groups. The results of the main outcomes are shown in Tables 2, 3, 4.

**TABLE 2:** Follow‐up results for LBP.

Low back pain	Outcome tool	Measure	Baseline		Pre‐surgery		Post‐surgery		3 weeks		1 month		8 weeks		3 months		6 months		1 year	
			Intervention	Control	Intervention	Control	Intervention	Control	Intervention	Control	Intervention	Control	Intervention	Control	Intervention	Control	Intervention	Control	Intervention	Control
Lindback et al.	VAS	Mean (SD)	56.0 (24.4)	59.7 (21.6)	−7.9 (2.3)	−3.4 (2.3)	NA		NA		NA		NA		NA		NA		−24.5 (3.0)	−31.8 (3.0)
Lotzke et al.	VAS	Mean (95% IC)	57.3 (51.7–62.9)	65.0 (61.0–68.9)	−1.0 (−5.8 to 3.7)	2.5 (−2.1 to 7.1)	NA		−13.6 (−21.1 to −6.1)	−19.6 (−27.2 to −12.0)	NA		−28.3 (−34.4 to −22.2)	−23.1 (−29.1 to −17.1)	−30.8 (−37.0 to −24.7)	−30.7 (−36.7 to −24.8)	−29.2 (−36.7 to −21.7)	−31.8 (−39.2 to −24.4)	NA	
Louw et al.	NPRS	Mean (SD)	4.57	5.12	4.44	5.12	NA		NA		2.09	3.39	NA		2.00	3.18	2.56	3.03	3.07	2.64
Nielsen et al.	VAS	Median (IQR)	45 (0–87)	54 (0–91)	41 (0–78)	53 (0–96)	31 (0–87)	21 (0–85)	NA		17 (0–50)	31 (0‐87)	NA		10 (0–93)	16 (0–81)	10 (0–69)	21 (0–62)	NA	
Rolving et al.	LBPRS	Median (IQR)	7.0 (5.3–8.0)	7.2 (6.0–8.0)	NA		NA		NA		NA		NA		−3.0 (−4.3 to −1.3)	−2.6 (−4.3 to −0.3)	−2.3 (−4.0 to −1.7)	−2.3 (−4.7 to −0.7)	−2.5 (−4.3 to −1.0)	−2.7 (−5.0 to −0.3)

Abbreviations: IC = interval of confidence; IQR = interquartile range; NA = not assessed; SD = standard deviations.

**TABLE 3:** Follow‐up results for leg pain.

Leg pain	Outcome tool	Measure	Baseline		Pre‐surgery		Post‐surgery		3 weeks		1 month		8 weeks		3 months		6 months		1 year	
			Intervention	Control	Intervention	Control	Intervention	Control	Intervention	Control	Intervention	Control	Intervention	Control	Intervention	Control	Intervention	Control	Intervention	Control
Lindback et al.	VAS	Mean (SD)	65.3 (22.1)	64.0 (20.0)	−10.5 (2.5)	−5 to 0 (2.5)	NA		NA		NA		NA		NA		NA		−35.0 (3.3)	−36.5 (3.3)
Lotzke et al.	VAS	Mean (95% IC)	37.3 (29.3–45.3)	33.5 (26.3–40.7)	3.0 (−10.9 to 17.0)	−1.3 (−15.1 to 12.4)	NA		−16.6 (−30.7 to −2.6)	−16.5 (−30.5 to −2.5)	NA		−19.4 (−32.7 to −6.0)	−19.0 (−32.2 to −5.8)	−24.4 (−37.2 to −11.6)	−26.2 (−38.8 to −13.6)	−17.6 (−30.9 to −4.2)	−21.6 (−34.8 to −8.4)	NA	
Louw et al.	NPRS	Mean (SD)	5.25	6.06	5.37	6.06	NA		NA		1.43	2.91	NA		1.96	2.82	2.44	2.79	1.63	2.73
Nielsen et al.	VAS	Median (IQR)	38 (0–95)	39 (0–94)	41 (0–78)	53 (0–96)	NA		NA		17 (0–50)	31 (0–87)	NA		10 (0–93)	16 (0–81)	10 (0–69)	21 (0–62)	NA	
Rolving et al.	LBPRS	Median (IQR)	7.0 (5.3–8.0)	7.2 (6.0–8.0)	NA		NA		NA		NA		NA		−3.2 (−5.3 to −1.3)	−2.3 (−4.7 to −0.3)	−2.8 (−5.0 to −1.3)	−2.0 (−5.7 to −0.3)	−2.8 (−4.7 to −1.3)	−1.3 (−6.0 to −0.3)

Abbreviations: IC = interval of confidence; IQR = interquartile range; NA = not assessed; SD = standard deviations.

**TABLE 4:** Follow‐up results for disability.

Disability	Outcome tool	Measure	Baseline		Pre‐surgery		Post‐surgery		3 weeks		1 month		8 weeks		3 months		6 months		1 year	
			Intervention	Control	Intervention	Control	Intervention	Control	Intervention	Control	Intervention	Control	Intervention	Control	Intervention	Control	Intervention	Control	Intervention	Control
Lindback et al.	ODI	Mean (SD)	65.3 (22.1)	64.0 (20.0)	−3.2 (1.1)	−0.6 (1.1)	NA		NA		NA		NA		−10.6 (1.7)	−14.0 (1.7)	NA		−15.0 (1.7)	−20.4 (1.7)
Lotzke et al.	ODI	Mean (95% IC)	35.7 (32.3–39.0)	38.0 (35.1–40.9)	0.8 (−3.7 to 5.3)	2.9 (−1.5 to 7.3)	NA		4.1 (−2.2 to 10.4)	2.3 (−3.9 to 8.5)	NA		−10.8 (−16.0 to −5.6)	−8.6 (−13.6 to −3.6)	−13.4 (−18.4 to −8.3)	−12.6 (−17.5 to −7.8)	−14.5 (−19.8 to −9.3)	−17.5 (−22.7 to −12.4)	NA	
Louw et al.	ODI	Mean (SD)	44.21	46.67	43.11	46.67	NA		NA		31.78	35–58	NA		20.81	29.15	23.33	24.48	24.15	23.58
Nielsen et al.	RMQ	Median (IQR)	16 (8–21)	17 (6–22)	14 (1–21)	17 (7–23)	NA		NA		12 (3–21)	17 (1–23)	NA		8 (0–20)	11 (0–22)	8 (0–20)	11 (0–23)	NA	
Rolving et al.	ODI	Median (IQR)	40.7 (13.2)	40.8 (15)	NA		NA		NA		NA		NA		−15 (−26 to −4)	1 (−14 to 8)	−18 (−24 to −7)	−4 (−16 to 4)	−14 (−26 to −5)	−6 (−26 to 4)

### 3.5 Secondary Outcome

Disability was assessed in all the included studies using tools such as ODI (Lindbäck et al. 2018; Lotzke et al. 2019; Louw et al. 2014; Rolving et al. 2015) and the RMQ (Nielsen et al. 2010). Three studies evaluated quality of life (Lindbäck et al. 2018; Lotzke et al. 2019; Nielsen et al. 2010), while two studies examined fear avoidance (Lindbäck et al. 2018; Rolving et al. 2015), catastrophizing (Lotzke et al. 2019; Rolving et al. 2015), anxiety and depression (Lindbäck et al. 2018; Lotzke et al. 2019), and self‐efficacy (Lindbäck et al. 2018; Lotzke et al. 2019). One study assessed kinesiophobia (Lotzke et al. 2019).

Three studies found significant differences in disability: one at 3 and 6 month follow‐ups, and two at pre‐surgery follow‐ups.

For the other secondary outcome, only a few significant differences were observed at specific follow‐up time points. Two studies reported significant improvements in quality of life: one at the pre‐surgery follow‐up (Lindbäck et al. 2018) and the other at the 1‐month follow‐up (Lotzke et al. 2019). Significant differences in fear avoidance were found in two studies, occurring at the pre‐surgery follow‐up (Lindbäck et al. 2018) and the 6‐month follow‐up (Rolving et al. 2015). Catastrophizing showed significant differences in one study at the 6‐month follow‐up (Rolving et al. 2015), while self‐efficacy improvements were reported in one study at the pre‐surgery follow‐up (Lindbäck et al. 2018). No significant differences were observed for anxiety and depression, or kinesiophobia.

### 3.6 Results of Synthesis

**FIGURE 3:**
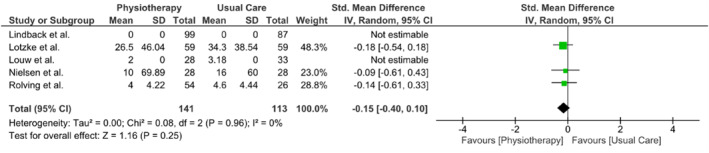
Meta‐analysis results of LBP at 3 months.

**FIGURE 4:**
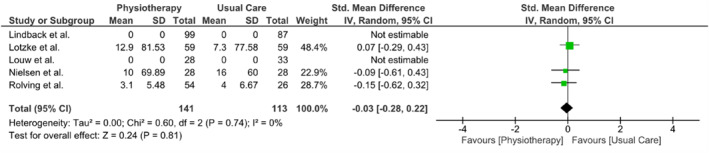
Meta‐analysis results of leg pain at 3 months.

**FIGURE 5:**
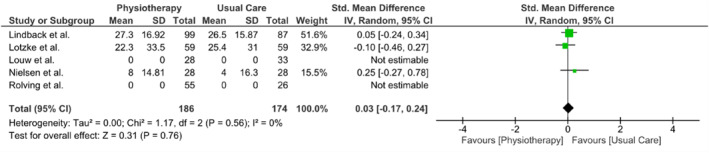
Meta‐analysis results of disability at 3 months.

Three meta‐analyses were performed to evaluate the outcomes of back pain, leg pain, and disability at the 3‐month follow‐up. Due to limited data availability from other studies, only three studies (Lindbäck et al. 2018; Lotzke et al. 2019; Nielsen et al. 2010; Rolving et al. 2015) were included in each meta‐analysis. All meta‐analyses showed no difference between interventions in any of the three outcomes. The results of the meta‐analyses are presented in Figures 3, 4, 5.

In two studies (Nielsen et al. 2010; Rolving et al. 2015), outcome data were presented as medians with interquartile ranges (IQRs), which preclude direct extractions of means and standard deviations for meta‐analysis. To this end, we assumed a normal distribution for these data so that the median equals the mean and calculated the standard deviation from the IQRs (SD = IQR/1.35). This assumption, which may introduce bias and should be acknowledged as a key limitation, allowed us to derive the required mean and SD values for inclusion of these studies in our pooled analyses.

### 3.7 Certainty of Evidence

The overall certainty of the evidence was assessed as very low according to the GRADE framework. This classification reflects the presence of a very serious risk of bias across all included studies. Additionally, the evidence was rated as serious for imprecision, which was influenced by a small sample size and imprecise confidence intervals. Details on the GRADE assessment, including the factors contributing to downgrading, are provided in Table 5.

**TABLE 5:** GRADE approach assessment.

Certainty assessment							N° of patients		Effect		Certainty
N° of studies	Study design	Risk of bias	Inconsistency	Indirectness	Imprecision	Other considerations	Preoperative physiotherapy	Usual care	Relative (95% CI)	Absolute (95% CI)	
Low back pain (follow‐up: 3 months; assessed with: VAS, NPRS, LBPRS)											
3	RCT	Very serious^a^	None	None	Serious^b^	None	141	113	//	−0.15 (−0.40, 0.10)	⊕◯◯◯
											Very low^c^
Leg pain (follow‐up: 3 months; assessed with: VAS, NPRS, LBPRS)											
3	RCT	Very serious^a^	None	None	Serious^b^	None	141	113	//	−0.03 (−0.28, 0.22)	⊕◯◯◯
											Very low^c^
Disability (follow‐up: 3 months; assessed with: ODI)											
3	RCT	Very serious^a^	None	None	Serious^b^	None	186	174	//	0.03 (−0.17, 0.24)	⊕◯◯◯
											Very low^c^

Abbreviations: CI, confidence interval; LBPRS, Low Back Pain Rating Scale; NPRS, Numeric Pain Rating Scale; ODI, Oswestry Disability Index; VAS, Visual Analogue Scale.

^a^ Downgraded two levels due to different biases in the measurement of the outcome.

^b^ Downgraded one level due to low sample sizes.

^c^ The GRADE approach uses different ⊕ to declare the level of certainty: one ⊕ means very low level of certainty (as in this review), two ⊕ means low, three ⊕ stands for moderate, four ⊕ stands for high.

## 4 Discussion

This systematic review analysed the efficacy of preoperative physiotherapy in patients with DDLS. From the pooled results, no difference was found between the preoperative intervention groups and the control groups in terms of low back pain, leg pain, and disability outcomes. However, the level of certainty was very low, and these results toward the potential benefit of preoperative physiotherapy in improving post‐surgical outcomes must be considered with caution.

In studies reporting preoperative improvements in the intervention group (Lindbäck et al. 2018; Nielsen et al. 2010), these effects were not sustained postoperatively, with no significant differences observed between exercise‐based or multimodal interventions and usual care.

These findings are consistent with the trial by Marchand, which was excluded from our review as the intervention was delivered by a kinesiologist. In their study, the intervention group showed improvements in leg pain only at the preoperative follow‐up (Marchand et al. 2021). These effects may not be sustained in the long term, and the benefits of surgery may outweigh those of pre‐surgery physiotherapy.

The interventions used in the studies included in this review ranged from cognitive‐educational therapy (e.g., CBT, PNE) (Lotzke et al. 2019; Louw et al. 2014; Rolving et al. 2015) to an exercise program (Nielsen et al. 2010) and a multimodal approach (Lindbäck et al. 2018).

A recent scoping review highlighted that, in the context of preoperative rehabilitation for spinal surgery, most studies primarily include cognitive‐educational therapies, while only 35% specifically focus on exercise‐based interventions, with considerable heterogeneity in the types of exercises used (Gränicher et al. 2025). Consistently, our review identified only one exercise‐based study (Nielsen et al. 2010) and another combining exercise with other interventions (Lindbäck et al. 2018).

Regarding cognitive‐educational therapy, two previous systematic reviews (Burgess et al. 2019; Janssen et al. 2021) found no significant improvements, in line with our findings. However, these reviews also included study designs other than RCTs and interventions not delivered by physiotherapists. Given the aim to assess intervention efficacy, it is generally preferable to include only randomized controlled trials (RCTs), as non‐randomized studies may introduce confounding and selection biases (Concato et al. 2000; Grimes and Schulz 2002).

In line with the findings of Gränicher et al. (2025), only Lindbäck and Nielsen investigated exercise‐based interventions in this review. However, several factors—such as treatment dosage and nutritional intake (Nielsen et al. 2010)—may have influenced the modest improvements observed at postoperative assessments. These factors could represent potential sources of performance bias affecting both the pre‐ and postoperative phases, limiting the ability to attribute the observed effects exclusively to preoperative physiotherapy.

Discrepancies were observed in the dosage and delivery of exercise interventions. For instance, Nielsen et al. implemented an individualised, home‐based exercise program, whereas participants in the study by Lindbäck, attended two supervised sessions per week over a 9‐week preoperative period (McConnell et al. 2024; Meuwissen et al. 2025). The dosage and delivery modalities of interventions may affect patients' adherence and the magnitude of the observed changes.

Furthermore, Rolving highlighted the importance of patient compliance, noting that some participants in the intervention group withdrew due to personal issues (e.g., lack of time) (Rolving et al. 2015). Lindback also considered compliance, reporting that 43% of patients did not reach the minimum number of required sessions (Lindbäck et al. 2018). The reasons for participants' withdrawal from the study were perhaps not reported.

Therefore, the limited effectiveness of preoperative physiotherapy reported in these studies may be partly explained by exercise‐related variables (e.g., quantity, type, frequency, intensity, etc.) as well as by patient compliance. In other surgical populations, such as the shoulder and lower limbs, preoperative programmes included structured and supervised exercise interventions and have reported improvements in postoperative functional outcomes (de Almeida et al. 2021; Kann et al. 2025; Punnoose et al. 2023; Shaarani et al. 2013). However, high heterogeneity across interventions has also been reported in other reviews. Kann et al. (2025) suggested that prehabilitation program durations and inconsistency in the delivery of multimodal approaches may have contributed to the heterogeneity observed in their review, while Punnoose et al. (2023) stated that heterogeneity reduced the certainty of evidence. This highlights the need for more structured and standardized intervention programs, which may help reduce heterogeneity and improve the robustness of future findings. Conclusions regarding prehabilitation effects across surgical sites should be interpreted cautiously, but these findings suggest that cognitive‐educational strategies alone may be insufficient to influence physical parameters such as muscle strength and functional capacity. The limited incorporation of adequately dosed exercise components in patients with DDLS may therefore have contributed to the absence of significant between‐group differences observed in this review.

The analysis of the included studies highlights that outcomes were generally reported in aggregate, without stratification by key characteristics such as age, gender, or the presence of comorbidities.

A systematic review found that preoperative sarcopenia appears to negatively affect postoperative pain, functional recovery, and quality of life (Chen et al. 2024). In a retrospective study including sarcopenic patients undergoing spinal surgery, Hirase reported that the group receiving preoperative physiotherapy had a shorter length of hospital stay, walked greater distances postoperatively, and experienced lower rates of total adverse events (Hirase et al. 2025). These findings suggest that an exercise‐based intervention tailored to patient's characteristics may enhance post‐operative outcomes.

Accordingly, investigating the effectiveness of preoperative physiotherapy through stratified analyses could be valuable. This would help identify whether its implementation in preoperative care is particularly beneficial for certain patient groups (e.g., elderly, frail, sarcopenic, etc.).

Different from the review conducted by Janssen in 2021 (Janssen et al. 2021), the present systematic review aimed to focus specifically on interventions delivered by physiotherapists. This decision was intended to support healthcare providers in better understanding the role of physiotherapists in the preoperative management of DDLS. As a result, some studies were excluded, including two RCTs in which the intervention was delivered by a kinesiologist (Marchand et al. 2021) and a physician (Saraçoğlu et al. 2021).

In the first one, Marchand et al. (2021) proposed an exercise program that showed improvements in disability and leg pain in the pre‐surgery follow‐up. In the other case, the PNE seems to be superior to usual care in kinesiophobia and disability in the 3‐month follow‐up. These findings are similar to those of this review.

Another significant limitation is the heterogeneity of the interventions and the generally low methodological quality of the included studies, which complicate comparisons between studies.

Finally, one author contacted for missing data or data presented using alternative statistical measures did not respond to our request.

### 4.1 Implications of Physiotherapy Practice

The current findings do not support preoperative physiotherapy as standard care for patients undergoing lumbar surgery for DDLS. Nevertheless, preoperative physiotherapy may improve pain and disability in selected patients, as its effectiveness is influenced by both intervention modalities and patient characteristics. In particular, frail or sarcopenic patients may benefit from a tailored‐exercise based intervention. Clinicians should decide whether to implement a preoperative physiotherapy program considering the individual characteristics.

## 5 Conclusion

The effects of preoperative physiotherapy, compared with usual care, on pain, disability, and hospitalisation among patients with DDLS, are minimal. However, the included studies were characterised by a high risk of bias, and the certainty of evidence, based on the GRADE approach, was very low. The heterogeneity in interventions may have reduced potential treatment effects and contributed to the absence of detectable differences between groups. There is a clear need for future high‐quality studies with low risk of bias that specifically investigate the effectiveness of preoperative physiotherapy and stratify patient populations to determine its efficacy in specific cohorts.

## 6 Practical Applications

- Current evidence is insufficient to support the systematic implementation of preoperative physiotherapy in patients undergoing surgery for DDLS.
- Most studies on preoperative physiotherapy focus predominantly on cognitive or educational interventions rather than exercise‐based approaches.
- There is a need for high‐quality research focusing on the effectiveness of preoperative physiotherapy in well‐defined patient populations, including elderly, frail, or sarcopenic individuals.

## Funding

The authors have nothing to report.

## Ethics Statement

The authors have nothing to report.

## Consent

The authors affirm that human research participants provided informed consent for publication, but we have no pictures or videos to declare.

## Conflicts of Interest

The authors declare no conflicts of interest.

## Data Deposition

The authors have nothing to report.

## Permission to Reproduce Material From Other Sources

The authors have nothing to report.

## Supporting information


Supporting Information S1

## Data Availability

The data that support the findings of this study are available from the corresponding author upon reasonable request.
