# Supplementary material for: The Added Value and the Efficacy of Preoperative Physiotherapy on Degenerative Diseases of the Lumbar Spine: A Systematic Review
Source: Physiother Res Int. 2026 Apr 17;31(2):e70214. doi: 10.1002/pri.70214 (PMC13088216; doi:10.1002/pri.70214)
Supplement: Supplementary file 1 — Supporting Information S1 [file PRI-31-e70214-s001.docx]

**MEDLINE**

"Intervertebral Disc Displacement"[MeSH Terms] OR ("Intervertebral Disc Displacement"[MeSH Terms] OR ("intervertebral"[All Fields] AND "disc"[All Fields] AND "displacement"[All Fields]) OR "Intervertebral Disc Displacement"[All Fields]) OR "Spinal Stenosis"[MeSH Terms] OR ("Spinal Stenosis"[MeSH Terms] OR ("spinal"[All Fields] AND "stenosis"[All Fields]) OR "Spinal Stenosis"[All Fields]) OR "Spondylitis"[MeSH Terms] OR ("Spondylitis"[MeSH Terms] OR "Spondylitis"[All Fields] OR "spondylitides"[All Fields]) OR "Spondylosis"[MeSH Terms] OR ("Spondylosis"[MeSH Terms] OR "Spondylosis"[All Fields]) OR "Intervertebral Disc Degeneration"[MeSH Terms] OR ("Intervertebral Disc Degeneration"[MeSH Terms] OR ("intervertebral"[All Fields] AND "disc"[All Fields] AND "degeneration"[All Fields]) OR "Intervertebral Disc Degeneration"[All Fields]) AND "Lumbosacral Region"[MeSH Terms] OR ("Lumbosacral Region"[MeSH Terms] OR ("lumbosacral"[All Fields] AND "region"[All Fields]) OR "Lumbosacral Region"[All Fields]) OR ("lumbarised"[All Fields] OR "lumbarization"[All Fields] OR "lumbarized"[All Fields] OR "lumbars"[All Fields] OR "Lumbosacral Region"[MeSH Terms] OR ("lumbosacral"[All Fields] AND "region"[All Fields]) OR "Lumbosacral Region"[All Fields] OR "lumbar"[All Fields]) AND "Preoperative Care"[MeSH Terms] OR ("Preoperative Care"[MeSH Terms] OR ("preoperative"[All Fields] AND "care"[All Fields]) OR "Preoperative Care"[All Fields]) OR ("preoperation"[All Fields] OR "preoperational"[All Fields] OR "preoperations"[All Fields] OR "preoperative"[All Fields] OR "preoperatively"[All Fields]) OR "Preoperative Period"[MeSH Terms] OR ("Preoperative Period"[MeSH Terms] OR ("preoperative"[All Fields] AND "period"[All Fields]) OR "Preoperative Period"[All Fields]) OR "Preoperative Exercise"[MeSH Terms] OR ("Preoperative Exercise"[MeSH Terms] OR ("preoperative"[All Fields] AND "exercise"[All Fields]) OR "Preoperative Exercise"[All Fields]) OR (("preoperation"[All Fields] OR "preoperational"[All Fields] OR "preoperations"[All Fields] OR "preoperative"[All Fields] OR "preoperatively"[All Fields]) AND ("physical therapy modalities"[MeSH Terms] OR ("physical"[All Fields] AND "therapy"[All Fields] AND "modalities"[All Fields]) OR "physical therapy modalities"[All Fields] OR "physiotherapies"[All Fields] OR "physiotherapy"[All Fields])) OR ("Preoperative Exercise"[MeSH Terms] OR ("preoperative"[All Fields] AND "exercise"[All Fields]) OR "Preoperative Exercise"[All Fields] OR ("preoperative"[All Fields] AND "rehabilitation"[All Fields]) OR "preoperative rehabilitation"[All Fields])

**CENTRAL**

#1 MeSH descriptor: [Spinal Stenosis] explode all trees

#2 MeSH descriptor: [Intervertebral Disc Displacement] explode all trees

#3 MeSH descriptor: [Intervertebral Disc Degeneration] explode all trees

#4 MeSH descriptor: [Spondylosis] explode all trees

#5 ("spinal stenosis"):ti,ab,kw

#6 ("intervertebral disc dispalcement"):ti,ab,kw

#7 ("intervertebral disc degeneration"):ti,ab,kw

#8 ("spinal disease"):ti,ab,kw

#9 ("spondylosis"):ti,ab,kw

#10 ("spondylolysis"):ti,ab,kw

#11 ("spondylolysthesis"):ti,ab,kw

#12 ("laminectomy"):ti,ab,kw

#13 MeSH descriptor: [Laminectomy] explode all trees

#14 ("diskectomy"):ti,ab,kw

#15 MeSH descriptor: [Diskectomy] explode all trees

#16 ("spinal surgery"):ti,ab,kw

#17 ("lumbar surgery"):ti,ab,kw

#18 #1 OR #2 OR #3 OR #4 OR #5 OR #6 OR #7 OR #8 OR #9 OR #10 OR #11 OR #12 OR #13 OR #14 OR #15 OR #16 OR #17

#19 MeSH descriptor: [Preoperative Period] explode all trees

#20 MeSH descriptor: [Preoperative Exercise] explode all trees

#21 ("preoperative period"):ti,ab,kw

#22 ("preoperative exercise"):ti,ab,kw

#23 ("preoperative rehabilitation"):ti,ab,kw

#24 ("prehabilitation"):ti,ab,kw

#25 ("preoperative physiotherapy"):ti,ab,kw

#26 ("preoperative physical therapy"):ti,ab,kw

#27 ("presurgery rehabilitation"):ti,ab,kw

#28 ("presurgery exercise"):ti,ab,kw

#29 ("preoperative training"):ti,ab,kw

#30 ("pain neuroscience education"):ti,ab,kw

#31 ("PNE"):ti,ab,kw

#32 ("cognitive functional therapy"):ti,ab,kw

#33 ("CFT"):ti,ab,kw

#34 ("cognitive behavioural therapy"):ti,ab,kw

#35 ("CBT"):ti,ab,kw

#36 ("acceptance and commitment therapy"):ti,ab,kw

#37 ("ACT"):ti,ab,kw

#38 MeSH descriptor: [Cognitive Behavioral Therapy] explode all trees

#39 #19 OR #20 OR #21 OR #22 OR #23 OR #24 OR #25 OR #26 OR #27 OR #28 OR #29 OR #30 OR #31 OR #32 OR #33 OR #34 OR #35 OR #36 OR #37 OR #38

#40 MeSH descriptor: [Lumbosacral Region] explode all trees

#41 ("lumbosacral"):ti,ab,kw

#42 ("lumbar"):ti,ab,kw

#43 #40 OR #41 OR #42

#44 #18 AND #39 AND #43

**PEDro**

Abstract & title:

1. surgery prehabilitation
2. preoperative

Body part: “lumbar spine, sacro-iliac joint or pelvis”

**Scopus**

TITLE-ABS-KEY

("spinal stenosis" OR "intervertebral disc displacement" OR "intervertebral disc degeneration" "intervertebral disc degeneration" OR spondylolysis OR spondylosis OR spondylolisthesis OR "spinal surgery" OR "lumbar surgery” OR diskectomy OR laminectomy OR "spinal fusion" OR decompression OR stabilization)

AND

("preoperative period" OR "presurgery period" OR "preoperative conditioning" OR "presurgery conditioning" OR "preoperative physiotherapy" OR "presurgery physiotherapy" OR "preoperative training" OR "presurgery training" OR "preoperative exercise" OR "presurgery exercise" OR prehabilitation OR "pain neuroscience education" OR pne OR "cognitive functional therapy" OR cft OR "cognitive behavioral therapy" OR cbt OR "acceptance and commitment therapy" OR act)

AND

("lumbar region" OR lumbar OR "lumbar spine" OR "lumbosacral region" OR lumbosacral OR "lumbar back")

**EMBASE**

('Intervertebral Disc Displacement'/exp OR ('Intervertebral Disc Displacement'/exp OR (intervertebral AND disc AND displacement) OR 'Intervertebral Disc Displacement') OR 'Spinal Stenosis'/exp OR ('Spinal Stenosis'/exp OR (spinal AND stenosis) OR 'Spinal Stenosis') OR Spondylitis/exp OR (Spondylitis/exp OR Spondylitis OR spondylitides) OR Spondylosis/exp OR (Spondylosis/exp OR Spondylosis) OR 'Intervertebral Disc Degeneration'/exp OR ('Intervertebral Disc Degeneration'/exp OR (intervertebral AND disc AND degeneration) OR 'Intervertebral Disc Degeneration')) AND ('Lumbosacral Region'/exp OR ('Lumbosacral Region'/exp OR (lumbosacral AND region) OR 'Lumbosacral Region') OR (lumbarised OR lumbarization OR lumbarized OR lumbars OR 'Lumbosacral Region'/exp OR (lumbosacral AND region) OR 'Lumbosacral Region' OR lumbar)) AND ('Preoperative Care'/exp OR ('Preoperative Care'/exp OR (preoperative AND care) OR 'Preoperative Care') OR (preoperation OR preoperational OR preoperations OR preoperative OR preoperatively) OR 'Preoperative Period'/exp OR ('Preoperative Period'/exp OR (preoperative AND period) OR 'Preoperative Period') OR 'Preoperative Exercise'/exp OR ('Preoperative Exercise'/exp OR (preoperative AND exercise) OR 'Preoperative Exercise') OR ((preoperation OR preoperational OR preoperations OR preoperative OR preoperatively) AND ('physical therapy modalities'/exp OR (physical AND therapy AND modalities) OR 'physical therapy modalities' OR physiotherapies OR physiotherapy)) OR ('Preoperative Exercise'/exp OR (preoperative AND exercise) OR 'Preoperative Exercise' OR (preoperative AND rehabilitation) OR 'preoperative rehabilitation'))
